# Cardiovascular and thromboembolic risk in ANCA-associated vasculitis: a nationwide matched cohort study by disease, sex and familial risk

**DOI:** 10.1093/rheumatology/keag467

**Published:** 2026-09-04

**Authors:** Hanna Lindberg, Klytaimnistra Voudouri, Lars Lindhagen, Ann Knight, Maria K Svensson, Johanna Dahlqvist

**Affiliations:** Department of Medical Sciences, Rheumatology, Uppsala University, Uppsala, Sweden; Department of Medical Sciences, Renal Medicine, Uppsala University, Uppsala, Sweden; Uppsala Clinical Research Center, Uppsala University, Uppsala, Sweden; Department of Medical Sciences, Rheumatology, Uppsala University, Uppsala, Sweden; Department of Medical Sciences, Renal Medicine, Uppsala University, Uppsala, Sweden; Uppsala Clinical Research Center, Uppsala University, Uppsala, Sweden; Department of Medical Sciences, Rheumatology, Uppsala University, Uppsala, Sweden

**Keywords:** ANCA-associated vasculitis, cardiovascular disease, thromboembolism, sex, familial factors

## Abstract

**Objectives:**

To investigate influence of sex, diagnosis (granulomatosis with polyangiitis (GPA) vs microscopic polyangiitis (MPA)) and familial factors on cardiovascular disease and thromboembolism (CVD–TE) risk in patients with ANCA-associated vasculitis (AAV).

**Methods:**

Adults with a diagnosis of GPA or MPA were identified from National Patient Registers (2005–2020) with five age- and sex-matched population controls randomly selected per patient. First-degree siblings of patients and matched controls were included. Outcomes were defined using ICD codes and included myocardial infarction (MI), ischaemic or haemorrhagic stroke, deep vein thrombosis (DVT), pulmonary embolism (PE), CVD–TE-related death and a composite CVD–TE end point. Hazard ratios (HRs) with 95% CIs were estimated using Cox proportional hazards models.

**Results:**

A total of 4317 AAV patients, 21 582 controls and 25 568 siblings were included. Both GPA and MPA were associated with increased CVD–TE risk compared with the controls (HR 2.04 [1.83, 2.38] and HR 2.45 [2.00, 2.99]), with no differences between GPA and MPA for individual outcomes. Both sexes had increased DVT and PE risks. In GPA, only males had increased MI risk (HR 2.25 [1.74, 2.92]), whereas only females had increased ischaemic stroke risk (HR 1.64 [1.25, 2.17]). Siblings of AAV patients had no excess CVD–TE risk. The highest CVD–TE risk occurred within 3 months of diagnosis (HR 7.69 [5.93, 9.99]).

**Conclusion:**

GPA and MPA were associated with comparable increased risks of CVD–TE, with the highest risk within 3 months following AAV diagnosis and with sex-specific differences in GPA. Lack of increased risk among siblings emphasize the vasculitis *per se* as a risk factor for CVD–TE.

Rheumatology key messages

- Both GPA and MPA are associated with substantially increased risk of cardiovascular and thromboembolic events.
- The risk of cardiovascular events differs between females and males with GPA.
- Findings highlight the early post-diagnostic period as critical for risk stratification.

## Introduction

ANCA-associated vasculitis (AAV) are rare but severe, systemic autoimmune disorders, comprising granulomatosis with polyangiitis (GPA), microscopic polyangiitis (MPA) and eosinophilic GPA (EGPA). The AAV are clinically heterogeneous, characterized by necrotizing inflammation of small- and medium-sized blood vessels affecting almost any organ and often leading to organ dysfunction [1]. EGPA is a distinct disorder characterized by eosinophilia and with a variable presence of ANCA. Given the differences in presentation and management, EGPA is not included in this study.

Historically, AAV were characterized by high mortality and poor long-term outcomes [2], but with advances in treatment and care, prognosis and overall survival have gradually improved [3, 4], leading to increased prevalence of both GPA and MPA [5, 6].

Despite reduced mortality, patients with AAV remain at elevated risk of comorbidities. Similar to many other autoimmune diseases [7], including RA [8, 9] and SLE [10, 11], AAV is associated with increased risk of cardiovascular disease and thromboembolism (CVD–TE) [12–15]. Multinational longitudinal cohorts report that 10–14% of patients experience at least one cardiovascular event within 5 years [16, 17], corresponding to a 2–4-fold risk of CVD–TE compared with a general population [18–22]. Reported events include myocardial infarction (MI) [22–24], deep vein thrombosis (DVT) [20, 21, 25, 26], pulmonary embolism (PE) [19, 21, 26, 27] and ischaemic stroke [13, 18, 28].

The increased CVD–TE risk in AAV is considered multifactorial, involving systemic inflammation, endothelial dysfunction [29, 30], disease-related chronic kidney disease (CKD) [16] and treatment-related factors such as glucocorticoid exposure [31, 32]. Patients with GPA exhibit evidence of accelerated atherosclerosis [33], possibly driven by persistent endothelial activation, oxidative stress and chronic low-grade inflammation [17, 34]. A genetic study has suggested a shared genetic predisposition for AAV and CVD–TE [35].

Previous studies have indicated that older age, disease severity, male sex and kidney involvement are associated with higher CVD–TE risk in AAV [16, 17, 20, 23]. However, it remains unclear whether risks vary by sex, differs between GPA and MPA, or is influenced by familial factors. Moreover, data on the risk of haemorrhagic stroke are limited.

The objective of this study was to determine the incidence, timing and patterns of CVD–TE in patients with GPA and MPA, stratified by sex and to assess whether first-degree relatives of patients with AAV have increased CVD–TE risk compared with relatives of matched population controls. To address these aims, we conducted a population-based matched cohort study using national healthcare registers in Sweden.

## Subjects and methods

### Data sources

The Swedish publicly funded healthcare system, together with the unique personal identity number assigned to each resident, enables linkage of national health and population registers. The National Patient Register (NPR) [36] includes hospitalizations since 1964 (with nearly 100% coverage since 1987), and specialist outpatient visits since 2001 (about 80% coverage) with diagnoses at inpatient discharge or outpatient visits recorded using ICD versions 7–10. The National Prescribed Drugs register contains information on all dispensations of prescribed drugs from Swedish pharmacies since 2005. However, the lack of information on drugs administered during inpatient care makes it unsuitable for in-depth analyses of AAV induction and maintenance treatment. The Register of Total Population at Statistics Sweden includes information on date of birth, sex, domicile, death and migration status for all residents. The Cause of Death Register includes dates and ICD-coded main and contributory causes of death. The Swedish Multi-Generation Register provides information on first-degree relatives of residents born in 1932 and later.

### Study population and index

During the last 25 years, development of classification criteria and refined testing have supported an increased sensitivity and specificity for the diagnosis of AAV. Therefore, the study period was defined as 1 January 2005 to 31 December 2020. All individuals with a diagnosis of GPA (ICD-10 M31.3) or MPA (ICD-10 M31.7) as a main or secondary diagnosis at discharge from inpatient care or at an outpatient visit within the study period, and aged 18 or more at diagnosis, were included. To include only newly diagnosed patients, individuals with a diagnosis of GPA or MPA recorded in the NPR prior to 2005 were excluded. Index date was set to the date of the first recorded GPA or MPA diagnosis.

To evaluate the accuracy of GPA and MPA diagnoses in the NPR, 10 patients per study year were randomly selected, adding up to 160 patients. This number was selected to ensure representation across the entire study period while maintaining a feasible sample size for detailed medical record validation. Access to sufficient material from medical records to perform a validation was granted for 123 patients (77%). An accurate diagnosis of GPA or MPA was confirmed in 96 (78%) of patients. Among misclassified cases, several patients were initially suspected of having AAV but were later reclassified with another diagnosis (e.g. polymyalgia rheumatica). A smaller number of patients were deemed clinically consistent with AAV but did not meet the classification criteria of the European Medicine Agency algorithm [37].

Using the Swedish Register of Total Population, five control individuals per patient (a commonly accepted control-to-case ratio to ensure statistical efficiency and precision of the estimates), matched for sex, age and county of residence at index date, were randomly selected. Controls were required not to have a diagnosis of GPA or MPA in NPR and inherited the index date from the matched patient. Patients and controls were linked to the Multi-Generation Register and first-degree siblings of patients and controls alive at index date were included. Siblings born before 1940 were excluded due to lack of mortality data before 1961. For comparative analyses of CVD–TE in patients vs their siblings, siblings inherited the index date from their corresponding patient (or from the day they turned 18 if they were younger at patient index). However, for comparative analyses of siblings to patients vs siblings to controls, we aimed to explore the risk of CVD–TE across the siblings’ lifetimes. Hence, in these analyses, index date for all siblings was set to the latter of 1 January 1987 (selected to ensure reliable NPR data) or the date of their 18th birthday. Siblings with an AAV diagnosis before index date were excluded.

Patients with AAV, population controls and siblings comprised the complete study population. Any individual who died on or before index date was excluded; if a patient was removed, their respective controls and/or siblings were also removed.

### Outcomes and baseline characteristics

Data on outcomes and co-morbidities at baseline were extracted for the study population from NPR. Outcomes of interest were MI, ischaemic stroke, haemorrhagic stroke, DVT and PE (see Table 1 for ICD codes). The events DVT and PE were considered only if the date of DVT/PE diagnosis was followed by a dispensation of an anticoagulant drug (ATC code B01A) recorded in the National Prescribed Drugs register within 30 days. The stricter definition of DVT and PE was chosen to improve specificity, even if some early fatal events may be missed. A sensitivity analysis to confirm the robustness of this approach was performed, analyzing risk of DVT and PE without the requirement of anticoagulant dispensation.

**Table 1:** ICD definitions of outcomes and co-morbidities.

Outcomes/co-morbidities	ICD10 (1997–)	ICD9 (1987–1996)	ICD8 (1969–1986)
Myocardial infarction	I210, I211, I212, I213, I219	410A, 410W, 410X	410,00, 410,99
Ischaemic stroke	I63	434	433,434
Haemorrhagic stroke	I61	431	431
Deep vein thrombosis	I80.1, I80.2	451B	451,00, 451,98, 451,99
Pulmonary embolism	I26	415B	450,01 450,02, 450,03, 450,09
Atrial fibrillation	I48	427D	427,92
Hypertension	I10, I11, I12, I13, I15	401, 402, 403, 404, 405	401, 402, 403, 404
Hyperlipidaemia	E78.0, E78.1, E78.2, E78.5	272A, 272B, 272C, 272E	272,00, 272,99
Diabetes mellitus	E10, E11	250	250
Chronic kidney disease	N18.0, N18.1, N18.2, N18.3, N18.4, N18.5, N18.8, N18.9	585	582,00, 582,09 583,99, 584,99
Kidney transplantation	Z94.0	V42A	KAS10, KAS20, 6670^a^
Dialysis	Z49.1, Z49.2, Z99.2	V56A, V56W	Y29,01

a Procedure codes.

Death due to MI, ischaemic or haemorrhagic stroke, DVT or PE was defined as a separate outcome (‘CVD–TE death’), with data extracted from the Cause of Death Register. The six outcomes were also combined into a composite outcome, CVD–TE (including all DVT and PE events).

Baseline comorbidities were defined as diagnoses recorded during the period from 10 years before until 180 days before the index date; although the 180-day cut-off may have resulted in the loss of some information on comorbidities, the restriction was set to reduce the larger risk of detection bias as patients tend to increase healthcare utilization near the index date. The comorbidities included MI, ischaemic and haemorrhagic stroke, DVT, PE, atrial fibrillation, hypertension, hyperlipidaemia and diabetes mellitus (Table 1). CKD and end-stage kidney disease (ESKD; including dialysis and kidney transplantation) were defined using ICD codes, and were recorded during the same period, as well as from 60 days before to 30 days after the index date to capture AAV-related CKD and ESKD (Table 1).

Retrieved data were pseudonymized with the key securely stored at Statistics Sweden. The study was approved by the Swedish Ethical Review Authority (Dnr 2021–00635, 2021–04920, 2022–06720-02, 2023–03751-02). Consent from individual patients was not required for the approvals.

### Statistical analyses

Cox proportional hazards models were used to estimate the hazard ratio (HR) with 95% CI of CVD–TE between the groups, using time since index as the time scale, and were illustrated using forest plots and cumulative hazard plots. Based on the exploratory nature of the study, the threshold of statistical significance was set at *P* < 0.05. To better estimate the risk of CVD–TE attributable to AAV-related inflammation and disease course, all analyses were adjusted for baseline comorbidities (MI, ischaemic and haemorrhagic stroke, DVT, PE, atrial fibrillation, hypertension, hyperlipidaemia and diabetes mellitus present before index; and CKD and ESKD present before or spanning index date), in addition to sex and age (unadjusted results are presented in Supplementary Tables S1–S4). Individuals were censored at death, emigration or end of follow-up. Siblings were censored as siblings if they received a diagnosis of GPA or MPA during follow-up. In two additional models, individuals were censored at 1 year and 90 days post-index date, respectively. Patients with GPA and MPA were analysed separately in comparisons with matched controls, including stratification by sex. The risk of CVD–TE was compared between patients with AAV and their unaffected siblings, as well as between siblings of patients with AAV and siblings of controls. Poisson models were used to estimate rate ratios (RRs) for the number of composite CVD–TE outcomes in AAV patients during the year and the 90-day period preceding the index date, respectively.

All performed tests were two-sided. Robust standard errors were applied to the Cox and Poisson models to account for clustering resulting from some individuals appearing multiple times in the dataset. For the Poisson model, this also handles overdispersion.

All analyses were performed in R (v.4.4.3; Vienna, Austria) using the packages survival (v.3.8–3), stats (v.4.3.3) and rms (v.7.0–0). No substantial *post hoc* changes were made to the original study plan. A completed STROBE checklist is provided in Supplementary Data S1.

## Results

### Clinical characteristics

A total of 4317 patients with AAV, 21 582 population controls and 25 568 siblings were included. Among patients with AAV, 3159 (73%) were diagnosed with GPA and 1158 (27%) with MPA. Baseline characteristics are summarized in Table 2.

**Table 2:** Baseline characteristics of the study population at index.

	Index date 1 January 2005					**Index date 1 January 1987** ^a^	
Baseline characteristic	AAV (*n* = 4317)	GPA (*n* = 3159)	MPA (*n* = 1158)	Siblings to patients (*n* = 4091)	Population controls (*n* = 21 582)	Siblings to patients (*n* = 4429)	Siblings to controls (*n* = 21 139)
Age, median years (min–max)	67 (18–100)	65 (18–93)	71 (18–100)	60 (18–81)	67 (18–101)	34 (18–47)	34 (18–47)
Males, *n* (%)	2147 (50)	1616 (51)	531 (46)	2056 (50)	10 734 (50)	2268 (51)	10 824 (51)
Follow-up, median years (min–max)	4.9 (0––16)	5.6 (0–16)	3.5 (0–15)	6.2 (0–16)	5.8 (0–16)	34 (0.1–34)	34 (0–34)
Follow-up, mean years (SD)	5.7 (4.4)	6.3 (4.5)	4.2 (3.6)	6.7 (4.4)	6.4 (4.3)	14.9 (3.1)	15.0 (3.0)
Myocardial infarction, *n* (%)	91 (2.1)	65 (2.1)	26 (2.2)	50 (1.2)	449 (2.1)	3 (0.1)	11 (0.1)
Ischaemic stroke, *n* (%)	149 (3.5)	106 (3.4)	43 (3.7)	59 (1.4)	639 (3.0)	2 (0.0)	6 (0.0)
Haemorrhagic stroke, *n* (%)	15 (0.3)	13 (0.4)	2 (0.2)	4 (0.1)	91 (0.4)	1 (0.0)	7 (0.0)
Deep vein thrombosis, *n* (%)	84 (1.9)	59 (1.9)	25 (2.2)	27 (0.7)	209 (1.0)	1 (0.0)	2 (0.0)
Pulmonary embolism, *n* (%)	92 (2.1)	54 (1.7)	38 (3.3)	19 (0.5)	201 (0.9)	1 (0.0)	2 (0.0)
Hypertension, *n* (%)	1120 (26)	661 (21)	459 (40)	539 (13)	4169 (19)	11 (0.2)	37 (0.2)
Atrial fibrillation, *n* (%)	373 (8.6)	226 (7.2)	147 (13)	140 (3.4)	1439 (6.7)	4 (0.1)	8 (0.04)
Hyperlipidaemia, *n* (%)	319 (7.4)	204 (6.5)	115 (9.9)	201 (4.9)	1261 (5.8)	0 (0.0)	1 (0.0)
Diabetes mellitus (type I & II), *n* (%)	385 (8.9)	237 (7.5)	148 (13)	220 (5.4)	1525 (7.1)	34 (0.8)	117 (0.6)
Chronic kidney disease, *n* (%)	368 (8.5)	150 (4.7)	218 (19)	28 (0.7)	220 (1.0)	4 (0.1)	12 (0.1)
End-stage kidney disease, *n* (%)	131 (3.0)	51 (1.6)	80 (6.9)	16 (0.4)	55 (0.3)	0 (0.0)	0 (0.0)

Comorbidities recorded during the period starting 10 years prior to index up to 180 days prior to index date.

a A separate index date (1 January 1987) was used for comparisons of siblings to patients with AAV to siblings of controls.

AAV: ANCA-associated vasculitis, GPA: granulomatosis with polyangiitis, MPA: microscopic polyangiitis.

At index, the median age of patients with AAV was 67 years, and 50% were male. The prevalence of pre-existing comorbidities was higher in patients with AAV than in the controls, including hypertension (26% vs 19%), CKD (8.5% vs 1.0%) and ESKD (3.0% vs 0.3%). Patients with MPA were older at diagnosis than patients with GPA (median age, 71 vs 65 years) and had a higher prevalence of hypertension (40% vs 21%), atrial fibrillation (13% vs 7.2%), diabetes mellitus (13% vs 7.5%) and CKD (19% vs 4.7%) at diagnosis of AAV (Table 2). During the period from 60 days before until 30 days after diagnosis of AAV, 338 (11%) GPA patients and 416 (36%) MPA patients developed CKD, and 144 (4.6%) GPA patients and 146 (13%) MPA patients developed ESKD, compared with <0.5% of controls and siblings (data not shown).

### Risk of CVD–TE in patients with GPA and MPA

**Figure 1:**
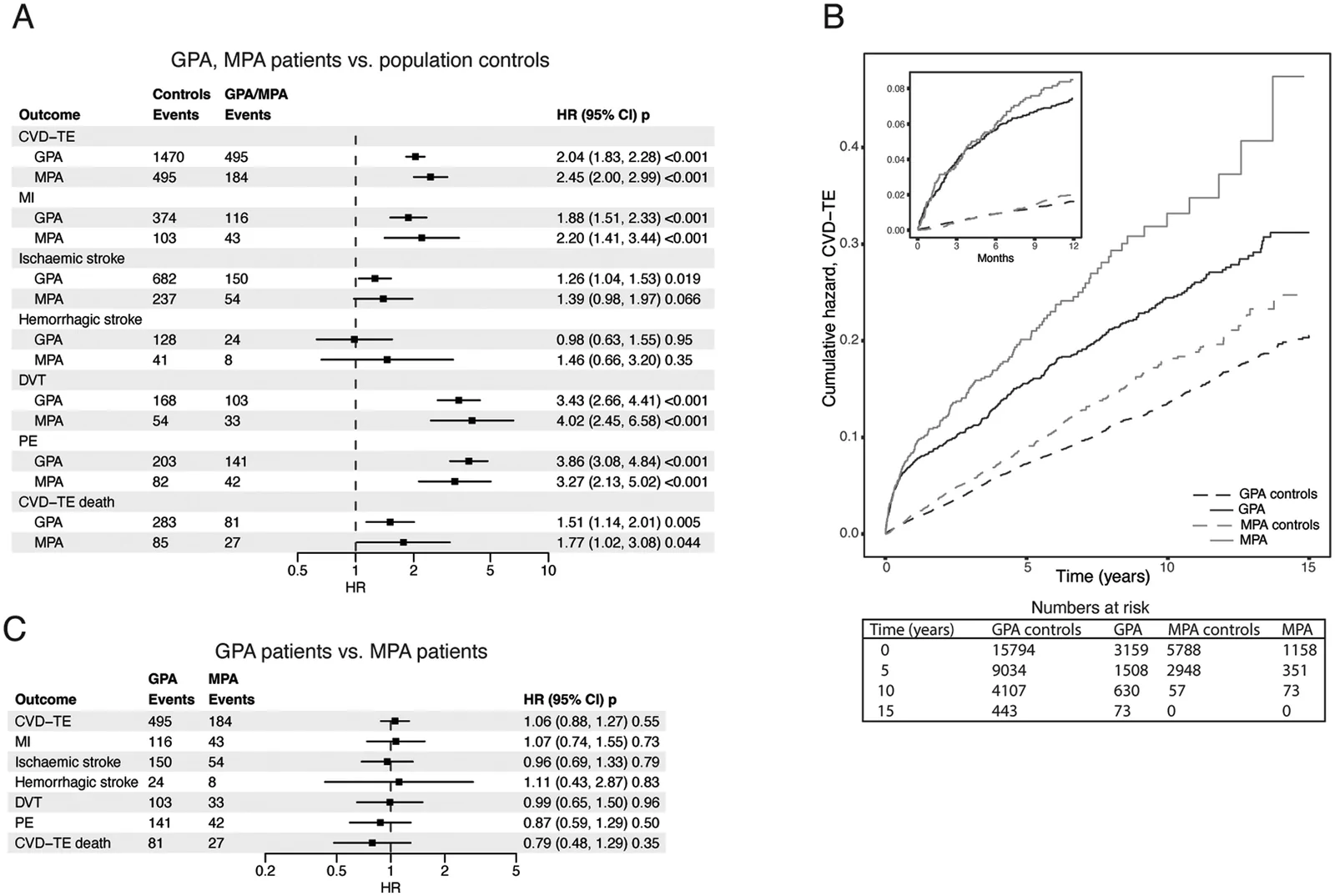
Risk of CVD–TE in patients with GPA and MPA. Forest plots (**A, C**) and cumulative hazard plot (**B**) illustrating the risk of six different CVD–TE outcomes and the composite CVD–TE outcome in patients with GPA and MPA in comparison with population controls (**A, B**) and in a direct comparison between GPA and MPA (**C**). GPA = granulomatosis with polyangiitis; MPA = microscopic polyangiitis; CVD–TE = cardiovascular disease and thromboembolism; MI = myocardial infarction; DVT = deep vein thrombosis; PE = pulmonary embolus; HR = hazard ratio; CI = confidence interval

Overall, both GPA and MPA were associated with an increased risk of CVD–TE compared with population controls (HR [95% CI] 2.04 [1.83, 2.28] and HR 2.45 [2.00, 2.99]). On average, there were 1.6 CVD–TE events per 10 patients among both the GPA and MPA groups, compared with 0.91 events per 10 controls, during the complete follow-up (Fig. 1A and B). Both GPA and MPA were associated with an elevated risk of MI (HR 1.88 [1.51, 2.33] and HR 2.20 [1.41, 3.44]), DVT (HR 3.43 [2.66, 4.41] and HR 4.02 [2.45, 6.58]), PE (HR 3.86 [3.08, 4.84] and HR 3.27 [2.13, 5.02]) and CVD–TE-related death (HR 1.51 [1.14, 2.01] and HR 1.77 [1.02, 3.08]; Fig. 1A). A sensitivity analysis of the hazard ratios for DVT and PE without the requirement of a dispensation of anticoagulant treatment yielded similar estimates (Supplementary Table S5). The risk of ischaemic stroke was increased in patients with GPA (HR 1.26 [1.04, 1.53]), but not in those with MPA. Neither GPA nor MPA was associated with an increased risk of haemorrhagic stroke compared with the controls, although the number of events was low.

A direct comparison between patients with GPA and patients with MPA revealed no significant difference in the risk of any CVD–TE outcome (Fig. 1C).

### Sex-specific risk of CVD–TE

**Figure 2:**
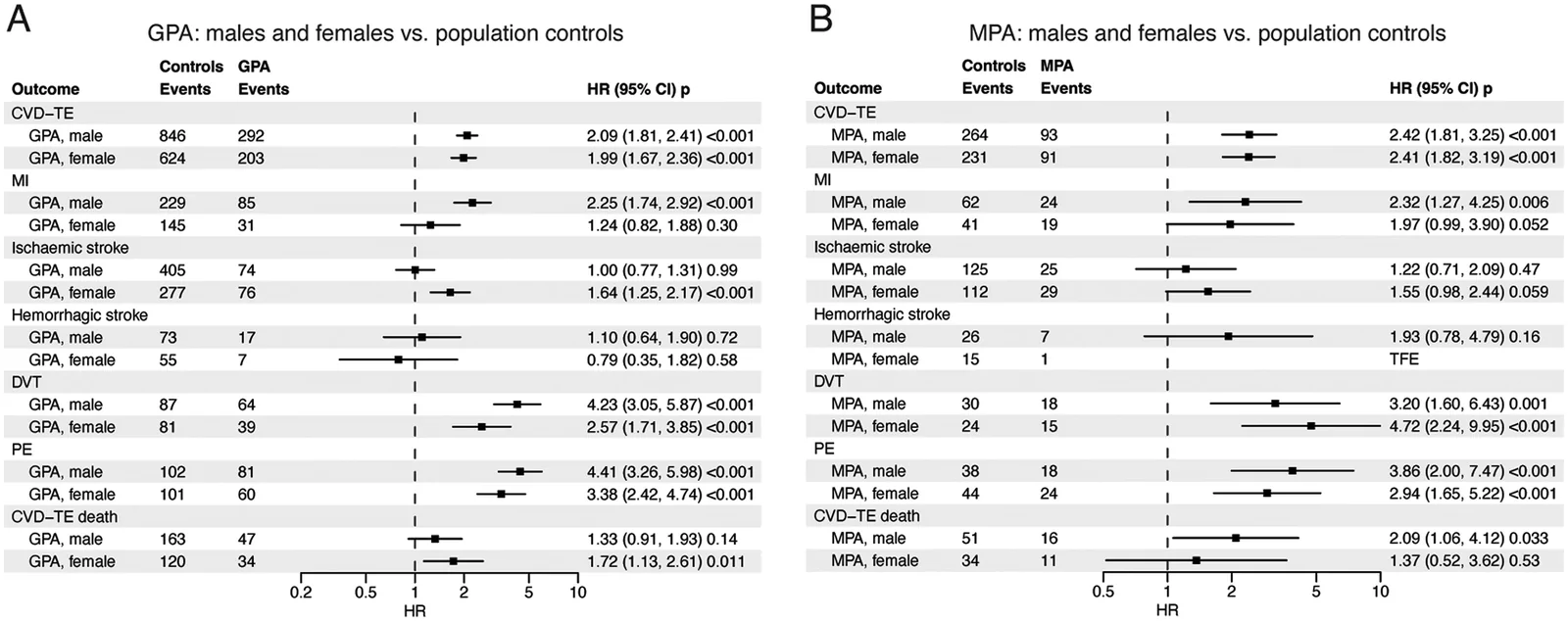
Risk of CVD–TE in males and females with GPA and MPA. Forest plot depicting the risk of six different CVD–TE outcomes and the composite CVD–TE outcome in patients with GPA and MPA in comparison with population controls, stratified by sex. GPA = granulomatosis with polyangiitis; MPA = microscopic polyangiitis; CVD–TE = cardiovascular disease and thromboembolism; MI = myocardial infarction; DVT = deep vein thrombosis; PE = pulmonary embolus; HR = hazard ratio; CI = confidence interval; TFE = too few events to allow analysis

Both males and females with GPA or MPA had a significantly increased risk of composite CVD–TE events compared with population controls (GPA: HR 2.09 [1.81, 2.41], HR 1.99 [1.67, 2.36] MPA: HR 2.42 [1.81, 3.25], HR 2.41 [1.82, 3.19]; Fig. 2A and B).

In patients with GPA, only males had an increased risk of MI (HR 2.25 [1.74, 2.92]), whereas only females had an increased risk of ischaemic stroke (HR 1.64 [1.25, 2.17]) and CVD–TE-related death (HR 1.72 [1.13, 2.61]; Fig. 2A). Both males and females had increased risks of DVT (HR 4.23 [3.05, 5.87] and HR 2.57 [1.71, 3.85]) and PE (HR 4.41 [3.26, 5.98] and HR 3.38 [2.42, 4.74]; Fig. 2A).

In patients with MPA, both males and females had increased risks of DVT (HR 3.20 [1.60, 6.43] and HR 4.72 [2.24, 9.95]) and PE (HR 3.86 [2.00, 7.47] and HR 2.94 [1.65, 5.22]), whereas the risk of ischaemic stroke was not increased for either sex. The risks of MI and CVD–TE-related death were increased in males (HR 2.32 [1.27, 4.25] and HR 2.09 [1.06, 4.12]), whereas females showed borderline increases in the risks of MI and ischaemic stroke (HR 1.97 [0.99–3.90] and HR 1.55 [0.98–2.44]; Fig. 2B).

Taken together, the increased risks of MI and ischaemic stroke appeared to be sex-specific among patients with GPA and MPA, whereas the risk for thromboembolic events was similar between the sexes. No increased risk of haemorrhagic stroke was observed in either group.

### Risk of CVD–TE in AAV siblings

**Figure 3:**
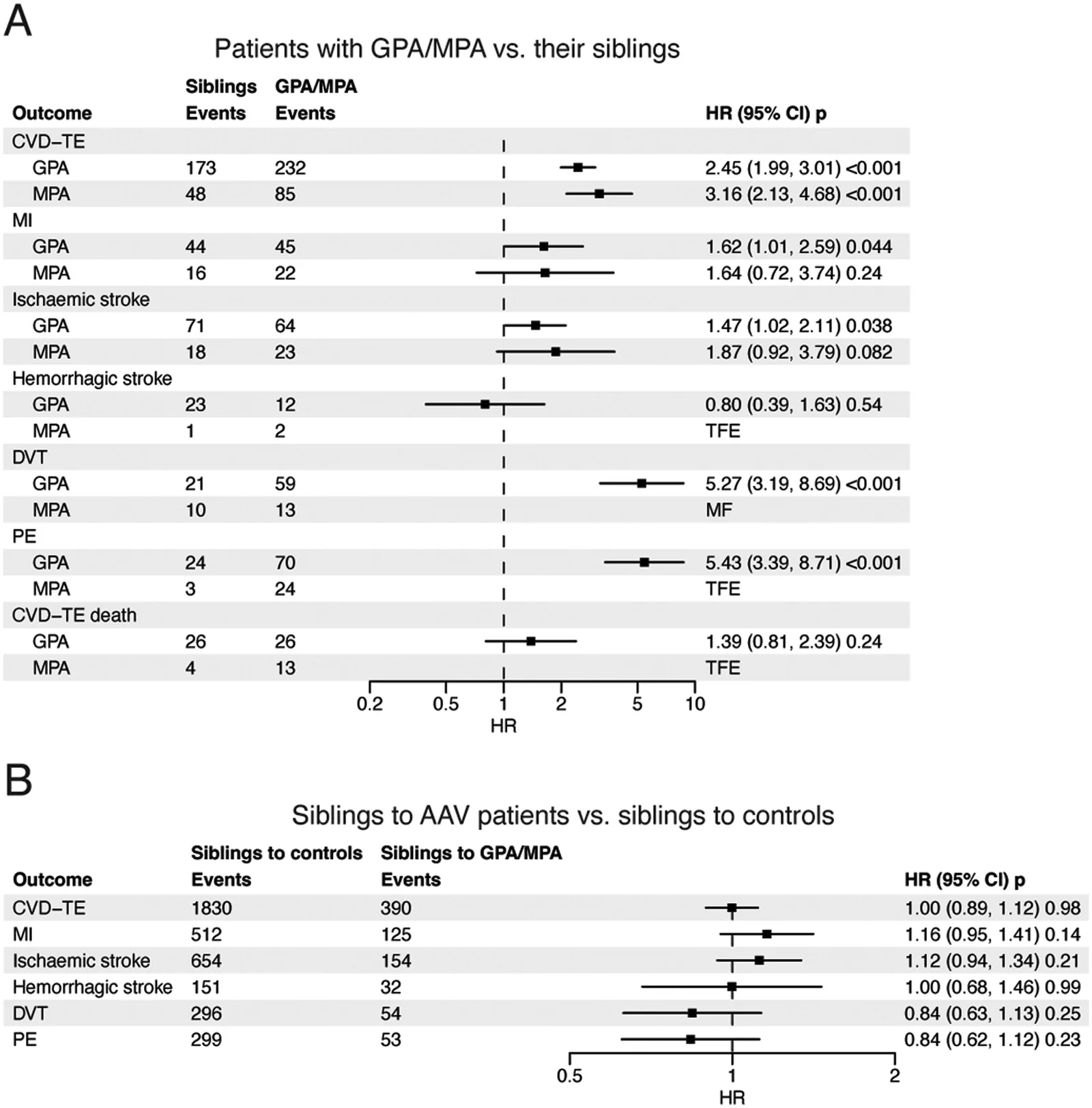
Risk of CVD–TE in siblings of patients with GPA and MPA. (**A**) Forest plot depicting the risk of six different CVD–TE outcomes and the composite CVD–TE outcome in patients with GPA and MPA in comparison with their siblings. (**B**) Forest plot illustrating the risk of CVD–TE outcomes in a comparison between siblings to AAV patients and siblings to population controls. AAV = ANCA associated vasculitis; GPA = granulomatosis with polyangiitis; MPA = microscopic polyangiitis; CVD–TE = cardiovascular disease and thromboembolism; MI = myocardial infarction; DVT = deep vein thrombosis; PE = pulmonary embolus; HR = hazard ratio; CI = confidence interval; TFE = too few events to allow analysis; MF = model failure—model failed to converge

To explore whether the increased risk of CVD–TE in patients with AAV is primarily driven by the vasculitis and the associated disease process, or whether there is a significant contribution from familial risk factors, we compared the risk of CVD–TE in patients to that in their siblings. Patients with GPA and MPA had overall increased risks of CVD–TE compared with their siblings (HR 2.45 [1.99, 3.01] and HR 3.16 [2.13, 4.68]; Fig. 3A). Unlike patients with MPA, patients with GPA had increased risks of MI (HR 1.62 [1.01, 2.59]) and ischaemic stroke (HR 1.47 [1.02, 2.11]) compared with their siblings, although the CIs indicated statistical uncertainty. In addition, patients with GPA had increased risks of DVT (HR 5.27 [3.19, 8.69]) and PE (HR 5.43 [3.39, 8.71]), whereas for MPA, the number of DVT and PE events verified by dispensation of anticoagulants was too small for analysis.

To further explore whether AAV and CVD–TE share familial susceptibility, we examined CVD–TE risk among siblings of AAV patients relative to siblings of matched population controls using register data from 1 January 1987 onwards. No increased risk of any CVD–TE outcome was observed in siblings of AAV patients compared with siblings of controls (Fig. 3B).

### Timing of CVD–TE events in AAV

**Figure 4:**
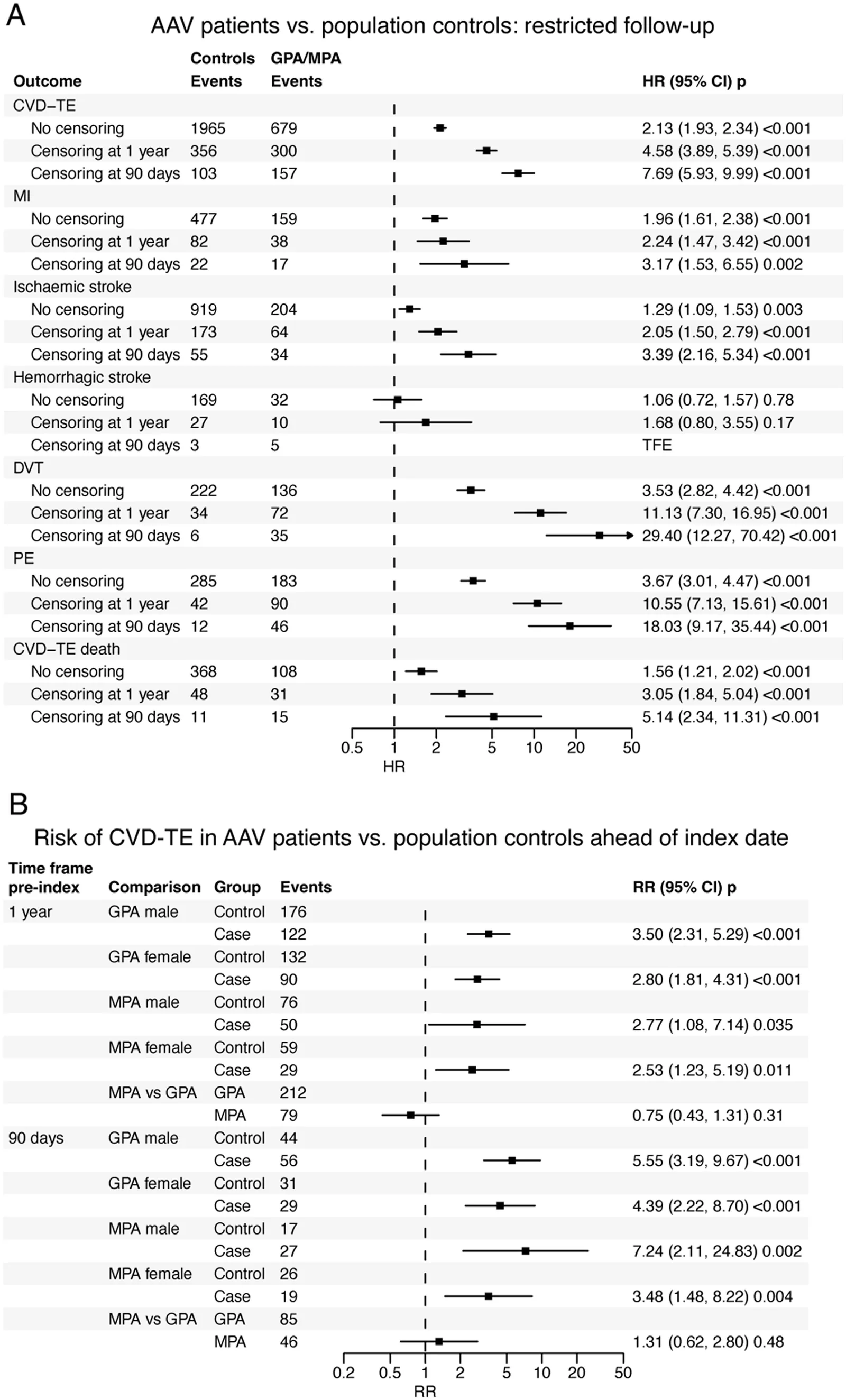
Risk of CVD–TE in patients with AAV the year prior to and after AAV diagnosis. (**A**) Forest plot depicting the risk of six different CVD–TE outcomes and the composite CVD–TE outcome in patients with AAV in comparison with population controls, with follow-up time censored after 1 year and 90 days after index date, respectively. (**B**) Forest plot illustrating the relative risk of the composite CVD–TE outcome in patients with AAV in comparison with population controls within 1 year and 90 days prior to index date, respectively. AAV = ANCA associated vasculitis; GPA = granulomatosis with polyangiitis; MPA = microscopic polyangiitis; CVD–TE = cardiovascular disease and thromboembolism; MI = myocardial infarction; DVT = deep vein thrombosis; PE = pulmonary embolus; HR = hazard ratio; CI = confidence interval; RR = relative risk; TFE = too few events to allow analysis

Cumulative hazard analysis demonstrated a marked accumulation of CVD–TE events during the first year following the index date in patients with AAV compared with population controls (Fig. 1B). To better characterize early risks, follow-up was censored at 1 year and at 90 days after the index date, respectively. The highest thromboembolic risks were observed during the first 90 days after diagnosis, substantially exceeding those observed across the overall follow-up period (DVT: HR 29.4 vs HR 3.53; PE: HR 18.0 vs HR 3.67). Similarly, risks of MI (HR 3.17 vs HR 1.96), ischaemic stroke (HR 3.39 vs HR 1.29) and CVD–TE-related death (HR 5.14 vs HR 1.56) were substantially higher in this early period (Fig. 4A). Moreover, the rate ratio for the composite CVD–TE outcome was increased among patients with AAV compared with controls during the year and the 90-day period preceding the index date, respectively (RR 3.06 [2.33, 4.00] and RR 5.04 [3.45, 7.37] data not shown), with comparable risks for GPA and MPA (Fig. 4B).

## Discussion

In this nationwide, population-based register study, we confirm previous findings showing that AAV are associated with increased risks of CVD–TE, even after adjustment for comorbidities and chronic kidney disease, which are traditionally associated with CVD–TE risk. Moreover, we identified sex-specific differences in CVD–TE risk, and for the first time, we evaluated the risk of CVD–TE in first-degree relatives of patients with AAV, exploring potential familial factors driving the increased CVD–TE risk.

Previous studies have reported an increased risk of MI, ischaemic stroke, DVT and PE in AAV overall, and in GPA [18, 21–23, 38]. For instance, Nygaard *et al.* demonstrated increased risks with HRs comparable to those observed in our study (MI HR 1.62; ischaemic stroke HR 1.58; DVT HR 3.13; PE HR 4.04) [24]. Few studies, however, have estimated the risk of CVD–TE separately in GPA and MPA or compared the risks between the diseases [21, 39]. Mourget *et al.* and Borgas *et al.* reported increased risks of MI and coronary artery disease among patients with MPA but not GPA [18, 23]. Mourget *et al.* demonstrated an increased risk of ischaemic stroke in both GPA and MPA, while Tabakovic *et al.* found no difference in the rate of ischaemic stroke between GPA and MPA [28]. In addition, Liapi *et al.* found no difference between GPA and MPA in venous thromboembolic event rates [26]. Overall, these studies have been limited by relatively small sample sizes.

In the present study with a substantially larger cohort, we were able to assess risks with robust statistical power and demonstrate that, after adjustment for age, sex, prior cardiovascular comorbidities and CKD, there were no significant differences in CVD–TE rates between GPA and MPA. Both GPA and MPA were individually associated with elevated risks of MI, DVT, PE and CVD–TE-related death in comparison with population controls. GPA, but not MPA, was associated with an increased risk of ischaemic stroke. However, this discrepancy may reflect limited statistical power in MPA due to the small number of stroke events. Taken together, despite the fact that GPA more frequently affects multiple organ systems, whereas kidney involvement is the predominant characteristic of MPA, there were few differences in risk of CVD–TE between the two AAV subtypes. Notably, we observed no increase in the risk of haemorrhagic stroke in either GPA or MPA, although the number of events was low, particularly in MPA.

In line with previous studies of different CVD–TE events, we observed the greatest relative increase in the risk of DVT and PE in both GPA and MPA [20, 21, 26, 40]. The incidence of DVT in AAV substantially exceeds that reported in other systemic rheumatic diseases, such as RA and SLE [40], suggesting that the vasculitis *per se* is of major mechanistic importance for these events, possibly as a result of an activated or dysregulated endothelium [41].

Cumulative hazard analyses demonstrated that CVD–TE events cluster early in disease, occurring predominantly within the first 90 days and the first year following AAV diagnosis, consistent with previous studies [27, 38]. However, a lower but sustained long-term risk also remains. Importantly, our findings corroborate data from a register-based study by Nygaard *et al.* [42], showing that CVD–TE risk increases already during the year preceding the clinical diagnosis of AAV. Together, these observations raise the possibility that subclinical vasculitis-related inflammation and endothelial injury may contribute to early atherosclerotic and thrombogenic processes prior to overt disease, or that shared pathobiological mechanisms underlie both AAV and CVD-TE. The lower but persistent long-term risk may reflect residual inflammation and flares, accumulated organ damage or treatment effects. However, the underlying drivers of the increased CVD–TE risk observed around the time of diagnosis, whether related to systemic inflammation, treatment exposure or heightened detection due to increased healthcare contact, cannot be determined in the present study, given the lack of detailed data on treatment and disease activity.

Sex-stratified analyses revealed important differences. In GPA, the risk of MI was doubled in males compared with the controls, with no significant increase in females. Conversely, females with GPA exhibited a 1.5-fold increased risk of ischaemic stroke and CVD–TE-related death, which was not observed in males, although the risk difference for CVD–TE-related death between the sexes was subtle. These findings are novel and suggest the presence of sex-specific determinants of CVD–TE risk beyond traditional cardiovascular risk factors in AAV. In MPA, the differences between the sexes were more subtle; additional independent studies are needed to dissect any plausible sex differences.

AAV patients demonstrated an increased risk of CVD–TE both relative to population controls and to their siblings. Siblings of patients exhibited a risk of CVD–TE comparable to that of siblings of population controls. Together, these findings argue against a major contribution of shared genetic risk factors of large effect between AAV and CVD–TE. However, given that complex diseases are typically influenced by multiple genetic variants of small effect, the sample size of the present study may have limited power to detect more modest shared genetic susceptibility. In addition, in hazard models without adjustment for sex, age and comorbidities (Supplementary Table S4), there was an increased risk of ischaemic stroke in siblings of patients with MPA compared with control siblings. In the context of all analyses conducted among case and control siblings, this isolated finding is hard to interpret. Overall, while our results do not support a substantial familial overlap in risk, a more subtle familial contribution cannot be excluded and additional studies addressing this issue are needed. To our knowledge, this is the first study to specifically assess familial contributions to CVD–TE risk in AAV.

The strengths of this study include its large sample size, which provided robust statistical power, minimized selection bias and enabled detailed analyses by AAV subtype and sex. The use of well-validated national registries ensured complete capture of the CVD–TE outcomes, with long-term follow-up. Thromboembolic endpoints were further validated through linkage with anticoagulant dispensation data. A particular strength of this study is the inclusion of sibling controls for both patients and population controls which allowed assessment of potential familial confounding.

There are limitations to this study. While Cox regression analyses can identify associations between exposures and outcomes, the observational nature of this study precludes any conclusions regarding causality. We cannot exclude confounding from factors such as smoking, disease activity, extent of organ involvement or medication. Lack of ANCA status hampered analyses stratified by ANCA subtype. Moreover, because siblings and controls had to be alive at the index date, the study may be subject to survival bias. DVT and PE events followed by death within 30 days would not be recorded because of the requirement of anticoagulant dispensation within 30 days requirement, possibly underestimating event rates for DVT and PE. Likewise, because of the identification of CKD based solely on ICD codes likely underestimates the number of individuals with kidney dysfunction. Misclassification of AAV diagnoses is possible due to reliance on ICD codes, though this is mitigated by the large sample size. Given the 78% validation rate for AAV diagnoses, the estimates may be biased towards the null, potentially leading to an underestimation of the true associations between AAV and CVD–TE risk. Time-varying Cox models were not applied, hence changes in risk factors over time may result in residual confounding. Finally, the cohort was predominantly of European ancestry, which may limit generalizability to other populations.

In conclusion, both GPA and MPA constitute strong and independent risk factors for CVD–TE, irrespective of pre-existing cardiovascular or kidney disease, with differences between the sexes in GPA. Notably, the CVD–TE risk begins to increase already during the year preceding the clinical diagnosis of AAV. This study highlights the importance of timely diagnosis and increased surveillance of both cardiovascular and thromboembolic events in patients with AAV.

## Supplementary Material

keag467_Supplementary_Data

## Data Availability

Data are not publicly available but are available from the corresponding author upon reasonable request.
