# Supplementary material for: Cardiovascular and thromboembolic risk in ANCA-associated vasculitis: a nationwide matched cohort study by disease, sex and familial risk
Source: Rheumatology (Oxford). 2026 Sep 4;65(9):keag467. doi: 10.1093/rheumatology/keag467 (PMC13581303; doi:10.1093/rheumatology/keag467)
Supplement: Supplementary file 1 [file keag467_supplementary_data.pdf]

Supplementary material

Cardiovascular and Thromboembolic Risk in ANCA-Associated Vasculitis: A Nationwide Matched Cohort Study by Disease, Sex, and Familial Risk

Authors:

Hanna Lindberg*[1], Klytaimnistra Voudouri*[2], Lars Lindhagen[3], Ann Knight[1], Maria K Svensson[2,3], Johanna Dahlqvist[1]

*Contributed equally.

Authors' affiliations:

[1]Dept. of Medical Sciences, Rheumatology, Uppsala University, Uppsala
[2] Dept. of Medical Sciences, Renal Medicine, Uppsala University, Uppsala
[3]Uppsala Clinical research Center, Uppsala University, Uppsala, all from Sweden

Supplementary Table 1. Unadjusted Cox proportional hazard analyses of patients with AAV/GPA/MPA in comparison with population controls, for six outcomes.

Supplementary Table 2. Unadjusted Cox proportional hazard analyses of patients with MPA in comparison with patients with GPA, for six outcomes.

Supplementary Table 3. Unadjusted Cox proportional hazard analyses of patients with GPA/MPA in comparison with their siblings, for six outcomes.

Supplementary Table 4. Unadjusted Cox proportional hazard analyses of siblings to patients with AAV/GPA/MPA in comparison with siblings to population controls, for six outcomes.

Supplementary Table 5. Sensitivity analysis: Cox proportional hazard analyses of risk of DVT and PE in patients with AAV/GPA/MPA in comparison with population controls.

Supplementary File 1. Strengthening the Reporting of Observational Studies in Epidemiology (STROBE) checklist.

Supplementary Table 1. Unadjusted Cox proportional hazard analyses of patients with AAV/GPA/MPA in comparison with population controls, for six outcomes.

	**HR (95% CI) p**								
**Outcome**	**All AAV. All follow-up**	**All AAV. Censoring after 1 year**	**All AAV. Censoring after 90 days**	**GPA. All follow-up**	**MPA. All follow-up**	**GPA female. All follow-up**	**GPA male. All follow-up**	**MPA female. All follow-up**	**MPA male. All follow-up**
CVD-TE	2.04 (1.87, 2.23) <0.001	4.57 (3.92, 5.33) <0.001	7.94 (6.19, 10.17) <0.001	1.97 (1.78, 2.18) <0.001	2.31 (1.94, 2.74) <0.001	1.87 (1.60, 2.20) <0.001	2.05 (1.79, 2.35) <0.001	2.38 (1.86, 3.04) <0.001	2.27 (1.79, 2.88) <0.001
MI	1.89 (1.58, 2.26) <0.001	2.44 (1.66, 3.59) <0.001	3.95 (2.10, 7.44) <0.001	1.74 (1.41, 2.14) <0.001	2.44 (1.71, 3.48) <0.001	1.19 (0.80, 1.75) 0.39	2.11 (1.65, 2.71) <0.001	2.63 (1.53, 4.54) <0.001	2.34 (1.47, 3.74) <0.001
Ischaemic stroke	1.25 (1.07, 1.45) 0.004	1.95 (1.47, 2.60) <0.001	3.18 (2.07, 4.87) <0.001	1.22 (1.02, 1.46) 0.028	1.35 (1.01, 1.82) 0.045	1.52 (1.18, 1.96) 0.001	1.01 (0.79, 1.30) 0.92	1.50 (1.00, 2.26) 0.052	1.23 (0.80, 1.88) 0.35
Hemorrhagic stroke	1.06 (0.73, 1.55) 0.76	1.95 (0.95, 4.03) 0.069	TFE	1.04 (0.67, 1.61) 0.86	1.14 (0.53, 2.42) 0.74	0.71 (0.32, 1.55) 0.38	1.29 (0.76, 2.19) 0.34	TFE	1.64 (0.71, 3.76) 0.25
DVT	3.47 (2.80, 4.28) <0.001	11.19 (7.46, 16.80) <0.001	29.96 (12.61, 71.17) <0.001	3.44 (2.70, 4.39) <0.001	3.57 (2.32, 5.49) <0.001	2.68 (1.83, 3.92) <0.001	4.17 (3.03, 5.75) <0.001	3.58 (1.89, 6.78) <0.001	3.62 (2.02, 6.50) <0.001
PE	3.66 (3.04, 4.40) <0.001	11.34 (7.87, 16.34) <0.001	19.76 (10.46, 37.31) <0.001	3.93 (3.17, 4.87) <0.001	3.01 (2.08, 4.36) <0.001	3.34 (2.43, 4.59) <0.001	4.51 (3.37, 6.03) <0.001	3.13 (1.91, 5.13) <0.001	2.87 (1.64, 5.02) <0.001
CVD-TE death	1.65 (1.33, 2.04) <0.001	3.40 (2.17, 5.34) <0.001	6.99 (3.21, 15.20) <0.001	1.59 (1.24, 2.04) <0.001	1.85 (1.20, 2.85) 0.005	1.55 (1.06, 2.27) 0.023	1.62 (1.17, 2.24) 0.004	1.83 (0.93, 3.61) 0.080	1.91 (1.09, 3.34) 0.024

CVD-TE: Cardiovascular disease-thromboembolism; MI:Myocardial infarction; DVT: Deep vein thrombosis; PE: pulmonary embolism; AAV: Anti-neutrophil cytoplasmic antibody-associated vasculitis; GPA: Granulomatosis with polyangiitis; MPA: microscopic polyangiitis; TFE: Too few events to allow analysis; HR: hazard ratio; CI: confidence interval.

Supplementary Table 2. Unadjusted Cox proportional hazard analyses of patients with MPA in comparison with patients with GPA, for six outcomes.

Outcome	HR (95% CI) p<br>All individuals
CVD-TE	1.31 (1.11, 1.56) 0.002
MI	1.48 (1.04, 2.10) 0.031
Ischaemic stroke	1.33 (0.97, 1.82) 0.077
Hemorrhagic stroke	1.31 (0.57, 3.01) 0.52
DVT	1.05 (0.70, 1.55) 0.82
PE	1.02 (0.72, 1.45) 0.90
CVD-TE death	1.68 (1.48, 1.90) <0.001

CVD-TE: Cardiovascular disease-thromboembolism; MI:Myocardial infarction; DVT: Deep vein thrombosis; PE: pulmonary embolism; GPA: Granulomato with polyangiitis; MPA: microscopic polyangiitis; HR: hazard ratio; CI: confidence interval.

Supplementary Table 3. Unadjusted Cox proportional hazard analyses of patients with GPA/MPA in comparison with their siblings, for six outcomes.

	**HR (95% CI) p**	
**Outcome**	**GPA**	**MPA**
CVD-TE	2.79 (2.29, 3.39) <0.001	3.73 (2.63, 5.29) <0.001
MI	2.03 (1.34, 3.08) <0.001	2.72 (1.44, 5.15) 0.002
Ischaemic stroke	1.80 (1.28, 2.52) <0.001	2.62 (1.42, 4.83) 0.002
Hemorrhagic stroke	1.03 (0.51, 2.08) 0.92	TFE
DVT	5.55 (3.39, 9.10) <0.001	2.61 (1.14, 5.95) 0.023
PE	5.84 (3.69, 9.26) <0.001	TFE
CVD-TE death	1.98 (1.16, 3.37) 0.012	TFE

CVD-TE: Cardiovascular disease-thromboembolism; MI:Myocardial infarction; DVT: Deep vein thrombosis; PE: pulmonary embolism;
GPA: Granulomatosis with polyangiitis; MPA: microscopic polyangiitis; TFE: Too few events to allow analysis; HR: hazard ratio; CI: confidence interval.

Supplementary Table 4. Unadjusted Cox proportional hazard analyses of of siblings to patients with AAV/GPA/MPA in comparison with siblings to population controls, for six outcomes.

	**HR (95% CI) p**		
**Outcome**	**All AAV**	**GPA**	**MPA**
CVD-TE	1.02 (0.91, 1.14) 0.75	0.97 (0.85, 1.10) 0.62	1.16 (0.95, 1.41) 0.15
MI	1.17 (0.96, 1.42) 0.13	1.12 (0.89, 1.42) 0.35	1.29 (0.90, 1.84) 0.17
Ischaemic stroke	1.13 (0.94, 1.34) 0.19	0.99 (0.80, 1.23) 0.95	1.52 (1.11, 2.08) 0.008
Hemorrhagic stroke	1.01 (0.69, 1.48) 0.96	1.22 (0.80, 1.84) 0.36	TFE
DVT	0.87 (0.65, 1.16) 0.34	0.84 (0.61, 1.16) 0.29	0.94 (0.57, 1.57) 0.82
PE	0.85 (0.63, 1.13) 0.26	0.78 (0.57, 1.07) 0.12	0.88 (0.49, 1.60) 0.68
CVD-TE death	---	---	---

CVD-TE: Cardiovascular disease-thromboembolism; MI:Myocardial infarction; DVT: Deep vein thrombosis; PE: pulmonary embolism; AAV: Anti-neutrophil cytoplasmic antibody-associated vasculitis; GPA: Granulomatosis with polyangiitis; MPA: microscopic polyangiitis; TFE: Too few events to allow analysis; HR: hazard ratio; CI: confidence interval.

Supplementary Table 5. Cox proportional hazard analyses of risk of DVT and PE in patients with AAV, GPA and MPA, respectively, in comparison with controls

	**HR (95% CI) p**	
**Outcome**	**GPA**	**MPA**
DVT	3.70 (2.94, 4.66) <0.001	4.54 (2.93, 7.04) <0.001
PE	3.89 (3.19, 4.73) <0.001	4.15 (2.91, 5.94) <0.001

Sensitivity analyses of risk of DVT and PE defined by ICD codes alone, not requiring dispensation of anticoagulant treatment. Analyses were adjusted for co-mordbidities, age, and sex. DVT: Deep vein thrombosis; PE: pulmonary embolism; AAV: Anti-neutrophil cytoplasmic antibody-associated vasculitis; GPA: Granulomatosis with polyangiitis; MPA: microscopic polyangiitis; HR: hazard ratio; CI: confidence interval.

STROBE Statement—checklist of items that should be included in reports of observational studies

Title: *Cardiovascular and Thromboembolic Risk in ANCA-Associated Vasculitis: A Nationwide Matched Cohort Study by Disease, Sex, and Familial Risk*

Item	Recommendation	Entry
**1a**	Indicate study design in title/abstract	"Nationwide matched cohort study" stated in Title (p.1).
**1b**	Informative abstract	Structured abstract including background, methods, results and conclusions (p.3).
**2**	Background/rationale	Scientific background and rationale described in Introduction (p.4-5).
**3**	Objectives	Study objectives and hypothesis stated in Introduction (p.5).
**4**	Study design	Nationwide register-based matched cohort study described in Subjects and Methods (p.5-6)
**5**	Setting	Data sources, study period and follow-up described in Subjects and Methods (p.5-7).
**6a**	Participants	Inclusion and exclusion criteria described in Subjects and Methods (p.5-7)
**6b**	Matching	Case-control matching described in Subjects and Methods (p.6)
**7**	Variables	Exposure (diagnosis of AAV), outcome (CVD-TE, definitions and ICD codes specified) and covariates defined in Subjects and Methods (p.5-7).
**8**	Data sources/measurement	Register sources and variable definitions described in Subjects and Methods (p.5-7).
**9**	Bias	Potential confounding addressed through multivariable adjustment. Misclassification bias minimized through use of validated national registers. Surveillance bias and residual confounding discussed. Subjects and Methods (p.5-8) and Discussion (p.11-14).
**10**	Study size	All eligible individuals identified in the national registers during the study period were included. No formal sample size calculation was performed. Subjects and Methods (p.5-8).
**11**	Quantitative variables	Handling of continuous and categorical variables described in Subjects and Methods (p.7-8).

**12a**	Statistical methods	Hazard ratios (HRs) with 95% confidence intervals (CIs) were estimated using Cox proportional hazards models. Adjustment variables specified. Poisson models applied for outcomes before index. Subjects and Methods - Statistical analyses (p.7-8).
**12b**	Subgroups/interactions	Stratified analyses described in Subjects and Methods - Statistical analyses (p.7-8).
**12c**	Missing data	Not applicable.
**12d**	Loss to follow-up	Censoring procedures described in Subjects and Methods - Statistical analyses (p.7-8).
**12e**	Sensitivity analyses	Sensitivity analysis described in Subjects and Methods (p.7) and Results (p.9) and presented in Supplementary Table 5.
**13a**	Participants	Eligibility criteria of participants of study are described in Subjects and Methods (p.5-6).
**13b**	Non-participation	Reasons for exclusion reported in Subjects and Methods (p.5-8).
**13c**	Flow diagram	Not applicable.
**14a**	Descriptive data	Baseline characteristics presented (Table 2).
**14b**	Missing data	Not applicable.
**14c**	Follow-up time	Follow-up reported for subgroups in Table 2.
**15**	Outcome data	Number of CVD-TE events and timing of event reported in Results (p.8-11), Figures 1-4.
**16a**	Main results	Adjusted HRs and relative risks (when applicable) with 95% CIs reported. (Figure 1-4).
**16b**	Category boundaries	Not applicable.
**16c**	Absolute risk	Results are reported and hazard ratios and relative risks, respectively, Results (p.8-11), Figures 1-4.
**17**	Other analyses	Subgroup analyses reported in Results (p.8-11).
**18**	Key results	Main findings summarized in Discussion (p.11-14).
**19**	Limitations	Study limitations discussed in Discussion (p.14).
**20**	Interpretation	Findings interpreted in light of previous studies. Discussion (p.11-14).
**21**	Generalizability	Generalizability discussed in Discussion (p.11-14).
**22**	Funding	Sources of funding and role of funders reported (p.15)
